# An unexpected case of Ramsay hunt syndrome: case report and literature review

**DOI:** 10.1186/1756-0500-6-337

**Published:** 2013-08-28

**Authors:** Mali Worme, Reena Chada, Lilia Lavallee

**Affiliations:** 1 3474 St. Famille, Apt 2, Montreal, Quebec H2X 2K8, Canada; 2 St. Clair Medical Centre, St Famille, Montreal, QC, Canada

**Keywords:** Ramsay hunt syndrome, Herpes zoster oticus, Cervical zoster, Corticosteroids, Antivirals

## Abstract

**Background:**

Ramsay Hunt Syndrome (RHS) is a rare, severe complication of varicella zoster virus (VZV) reactivation in the geniculate ganglion. Facial paralysis is one of the features and without treatment, it fully recovers in as little as 20% of cases; this is much improved if treatment is started within 72 hours. This case is noteworthy in that coexistence of facial palsy with cervical dermatome involvement by VZV is not typical of RHS. Yet, it has been reported before.

**Case presentation:**

A 54 year old Caucasian woman presented with right ear discomfort, difficulty hearing and a vesicular rash along the pinnae, 8 days after the eruption of a similar rash in the right C2-C4 dermatomes. 2 days later, she awoke with a partial right-sided facial paralysis, which improved with treatment (valacyclovir and prednisone).

**Conclusions:**

This case is most pertinent to Family Practice, Otolaryngology and Neurology. It highlights the possible co-existence of RHS with cervical VZV reactivation and encourages physicians to monitor for this complication even before geniculate ganglion reactivation occurs. RHS is a rare disease that can present with vague symptoms. A high index of suspicion and close follow up are essential. Early intervention with antivirals and corticosteroids has shown significantly improved outcomes in these patients.

## Background

Ramsay Hunt Syndrome (RHS), also called Herpes Zoster Oticus, is a rare, severe complication of varicella zoster virus (VZV) reactivation. The classic triad consists of otalgia, vesicles in the auditory canal and ipsilateral facial paralysis [1]*.* Without treatment, full recovery of the facial paralysis occurs in as little at 20% of cases; this is much improved if treatment is started within 72 hours [2]. The risk of RHS in VZV reactivation has been quoted as little as 0.2% at day 60 [3]. However, the long-term morbidity that may result from RHS makes this an important topic of discussion. This case is noteworthy in that coexistence of facial palsy with cervical dermatome involvement of VZV is not typical of RHS. The aim of this article is to inform physicians about the presentation, including atypical, and management of RHS in order to facilitate prompt diagnosis, treatment and appropriate specialist referral.

## Case presentation

**Figure 1:**
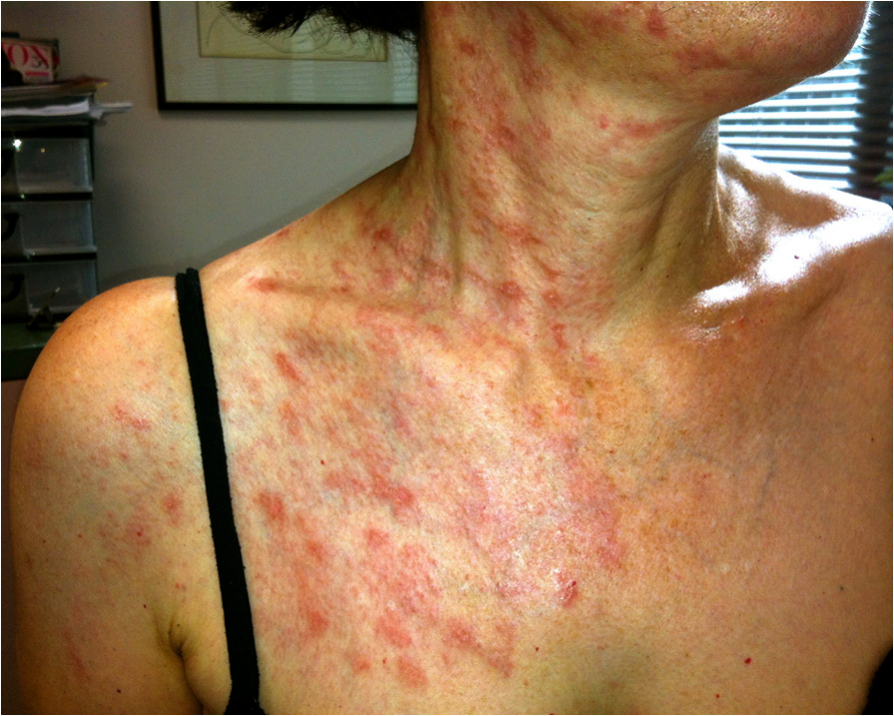
The sharp midline delineation of the patient’s rash on day 24.

A 54-year-old Caucasian female, with no past medical history, developed right-sided neck pain on day 1. On day 4, an erythematous, vesicular rash erupted at the site of pain. She was diagnosed with contact dermatitis at a walk-in clinic. Over days 4–9, she developed a right earache and headache and the rash spread to her upper right chest-wall. The rash was described as “dull and achy with intermittent sharp pain”. On day 9, at the family doctor’s office, exam showed an erythematous, vesicular eruption on the right anterior chest, neck and shoulder (C2, C3 and C4 dermatomes). The rash halted at the midline (Figure 1). It was tender, warm and blanched when palpated. The patient was diagnosed with herpes zoster (HZ) and secondary cellulitis. Valacyclovir, cephalexin and acetaminophen were started. On day 12, she returned with right ear discomfort and difficulty hearing. Exam showed a similar rash along the auricle but the external auditory canal was normal. Nortryptyline was started for neuralgia. On day 14, she woke up with partial right-sided facial paralysis. In the Emergency Room, RHS was diagnosed. Valacyclovir was extended to 10 days and prednisone started. A neuro-ophthalmology referral was made. On day 46, the patient returned showing significant improvement. She could smile symmetrically and close her right eye completely.

### Epidemiology of RHS

The Center for Disease control estimates that 32% of people in the USA will have HZ during their lifetime [4]. The most important risk factor is increasing age. RHS has a much lower incidence, presenting in only 0.2% of all HZ cases [3,5]. After primary VZV infection, the virus can remain dormant in sensory dorsal root ganglia. Reactivation causes HZ (shingles) - a painful, ipsilateral, vesicular eruption in a limited dermatomal distribution. Pain may precede the vesicles. In RHS, VZV reactivates in the geniculate ganglion, causing peripheral facial paralysis, otalgia and auricular vesicles [1]. Late diagnosis is common. The vesicles may appear after facial paralysis in a minority of cases (14% of cases in the only prospective study done) [6]. This means that a proportion of patients diagnosed with Bell’s palsy actually have RHS sine herpete. Cervical involvement of herpes zoster is rare with RHS and therefore makes this case one of special interest. As RHS is a reactivation of VZV in the geniculate nucleus, the etiology of co-existent cervical dermatome involvement is not well understood. Three theories have been hypothesized [7]. One such theory is that of cerebrospinal fluid (CSF)/hematogenous spread. In one study, pleocytosis in the CSF was seen in 38% of cutaneous herpes zoster cases [8]. Moreover, *Haanpaa* et al. isolated VZV in the CSF of 21% of patients with cutaneous lesions, but without meningeal or encephalitis symptoms [9]. Additionally, viremia is a common occurrence during zoster [10]. Since VZV has been documented in both blood and CSF during herpes zoster, these routes of spread could account for co-existent multi-dermatome involvement in HZ. Another theory is interneuronal communication. Brown et al. have shown that the second to fourth cervical nerves of the cervical plexus interconnect with peripheral branches of the facial nerve in some individuals [11]. Numerous interconnections between the lower cranial nerves and upper cervical nerves have also been illustrated. These linkages are highly variable amongst individuals [12]. It is plausible that VZV and associated inflammation may spread from a primary site of reactivation, through these anastomoses. Finally, the simultaneous activation of multiple ganglia may occur and could account for this clinical picture. Physicians should assess for immunocompromised states when patients present with multi-dermatomal VZV reactivation.

### Diagnosis of RHS

**Figure 2:**
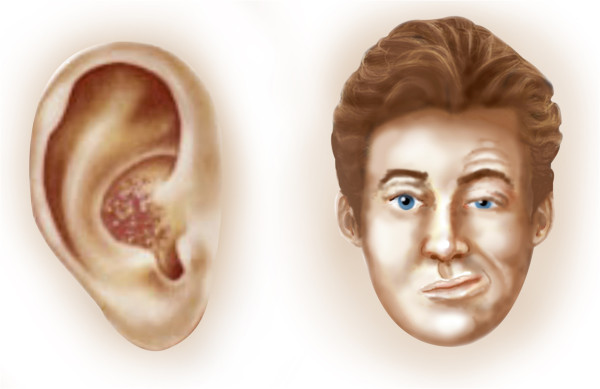
RHS signs: vesicular lesions in the ear, facial drooping, widened palpebral fissure and decreased smile.

Ramsay Hunt Syndrome is diagnosed clinically and is based on unilateral facial weakness plus vesicular lesions in the ipsilateral ear, hard palate or anterior 2/3 of the tongue [6]. Facial weakness is identified by facial drooping, a widened palpebral fissure and decreased smile on the affected side (Figure 2). The associated pain is described as dull and aching with associated allodynia. Otalgia or vertigo completes the triad picture of RHS. The individual symptoms of RHS are non-specific and can be seen in other diseases; the differential is described in Table 1. Some 2-23% of unilateral facial palsies without vesicles are actually herpes zoster sine herpete [13]. Therefore lesions are not required for diagnosis of RHS [6]. The traditional diagnostic triad of RHS cannot be applied to the atypical presentations of RHS, including multiple nerve involvement as seen in this case [14]. The use of CSF analysis or MRI adds no additional diagnostic value [15]. Finally, the gold standard for diagnosing VZV reactivation is polymerase chain reaction of skin, saliva or middle ear fluid samples but this is rarely done clinically [16].

**Table 1:** Differential diagnosis of Ramsay hunt syndrome

**Differential diagnosis**	**Features**
1/Bell’s palsy	Bell’s palsy is a diagnosis of exclusion for unilateral facial weakness [17]. Erythematous vesicular rash and otalgia suggest Ramsay Hunt.
2/Otitis externa	The ear pain in RHS can be mistaken for otitis [18]. Onset of rash and development of facial palsy differentiate Ramsay Hunt from otitis.
3/Trigeminal neuralgia	Trigeminal neuralgia pain is similar to Ramsay Hunt but tends to be paroxysmal and stimulated by triggers. It is not associated with any skin manifestations or neurological losses [19].

### Complications of RHS

1. Corneal abrasions and ulcers, if eye-lid closure is impaired

2. Secondary infection with bacteria (cellulitis)

3. Postherpetic neuralgia, as with other Varicella Zoster virus reactivations

4. Permanent facial paralysis

5. Long term ipsilateral hearing loss and tinnitus

### Management of RHS

Pharmacologic treatment of HZ complicated by RHS is controversial and requires ongoing research. Antivirals and corticosteroids are the current mainstay of treatment. Acyclovir, valacyclovir and famcyclovir have been shown to reduce the duration of acute HZ symptoms and associated long-term nerve damage. These drugs are well tolerated and are Food and Drug Administration (FDA) approved first line therapies for HZ [20]. They are also used in RHS. In addition, the potent anti-inflammatory effect of steroids has been argued to enhance recovery in RHS by reducing the inflammation and edema of the facial nerve, thus reducing damage [21].

### Steroids

There is a paucity of published data on the efficacy of corticosteroids in RHS specifically. A Cochrane Review found no randomized controlled trials investigating the use of corticosteroids as in Ramsay Hunt syndrome [22]. Yet, large RCTs showed that adjunct corticosteroids resulted in quicker healing of the rash and decreased incidence and severity of pain during HZ. Yet, there was no reduction in the incidence or severity of post herpetic neuralgia in these studies [20,23].

### Steroids and antivirals

A Cochrane Review of the sole randomized controlled trial (RCT) comparing treatment with antivirals and corticosteroids to corticosteroids alone in 15 RHS patients showed no significant difference in outcomes [24]. A subsequent meta-analysis of 12 RHS articles concluded that antiviral therapy plus steroids compared to steroids alone significantly improved facial nerve function recovery (odds ratio of 2.8, 95% Confidence Interval (CI)) [25].

The largest RHS treatment study was a retrospective analysis of 80 cases. Patients treated with acyclovir and prednisone within 72 hours of symptom onset had a complete recovery rate of 75% vs those treated after 7 days, who had a complete recovery rate of 30% [16]. In addition, 50% of patients who were not treated in the first 3 days went on to have a complete loss of response to facial nerve stimulation.

### Adjuvant treatment

Eye patches, taping the eye closed, artificial tears and oral analgesics are also used in the management of RHS.

Despite the absence of RCTs on RHS management, when considering the possibility of lifelong facial paralysis and hearing loss, experts recommend combination antiviral and corticosteroid therapy within the first 72 h of symptoms [6,16]. Though no set-dosing regimen exists, 800 mg acyclovir 5 times/day for 7–10 days and Prednisone 1 mg/kg for 5 days and taper is used in published trials. In our case, acyclovir was replaced by 1 g valacyclovir TID for 10 days, with successful outcomes.

## Conclusions

Ramsay Hunt syndrome is a rare disease that can present with vague symptoms and atypical presentations as seen in this case with cervical dermatome involvement. A high index of suspicion and close follow up are essential in patients who have HZ symptoms. Early intervention with antivirals and corticosteroids has shown to significantly improve outcomes in these patients.

## Consent

Written informed consent was obtained from the patient for publication of this Case report and any accompanying images. A copy of the written consent is attached, for review by the Editor of this journal.

## Abbreviations

CI: Confidence interval; CSF: Cerebrospinal fluid; FDA: Food and drug administration; HZ: Herpes zoster; RCT: Randomized controlled trial; RHS: Ramsay hunt syndrome; TID: Three times per day; VZV: Varicella zoster virus.

## Competing interests

The authors declare that they have no competing interest.

## Authors’ contributions

(MW) assessed the patient and decided to create a case report. She did a literature review, analyzed and interpreted the data, drafted and revised the manuscript. (RC) assessed and treated the patient in the family doctor’s office, acquired the data from the various clinics and emergency rooms and contributed to the manuscript drafting. (LL) did a literature review, analyzed and interpreted the data, contributed to drafting and revised the manuscript. All authors read and approved the final manuscript.

## Authors information

Mali Worme is a fourth year medical student at McGill University in Montreal, Quebec. She completed her Bachelor’s degree in Physiology and International Development Studies in 2010 at McGill University.

Dr Reena Chada is a family physician practicing in mid-town Toronto, Ontario. She completed her undergraduate medical training at the Michael G. DeGroote School of Medicine at McMaster University in Hamilton, Ontario followed by a residency program in Family Medicine at the University of Toronto in Toronto, Ontario.

Lilia Lavallee is a fourth year medical student at McGill University in Montreal, Quebec. She completed her Bachelor’s degree in Physiology in 2010 at McGill University.
